# 

**Published:** 1919

**Authors:** 


### SUBJECT INDEX

ABBREVIATIONS :

A. = Abstract. R. = Research Society Report.

C. = Circular. I. A. C. = Interallied Conference.

E. = Editorial. I. S. C. = Interrallied Surgical Conference.

**Abdomen,** Surgery. Churchman. **A.**, 853.
Surgery. Richards. **A.**, 78.
Traumatic. Deaver. **A.**, 1084.
**Abscess,** Lungs. Gregoire. **A.**, 370.
**Acid,** Trichloracetic. Gallaher. **A.**, 1409.
Carbonic. Shaw-Mackenzie. **A.**, 645.
**Acidosis. C.**, 138.
Desplas. **A.**, 1086.
**Adhesive,** for Traction. Cunningham. **A.**, 208.
**Agglutination Test.** Broughton-Alcock. **A.**, 393.
**Alkali**, Action on Wounds. Flessinger and Clogne. **A.**, 360.
**Ambulances.** James. **R.**, 199.
**Amputation**. **R.**, 90.
Putti. **A.**, 376.
Gaudeans. **A.**, 378.
Phocus. **A.**, 875.
Cinematis. Baer and Wilson. **A.**, 218.
**Anesthesia.** Barlow. **A.**, 1144.
Bureau. **A.**, 1054.
Chaput. **A.**, 842.
Lapeyre. **A.**, 841.
Experiments. Gwathmey and Tibbits. **A.**, 1057.
Gas Poisoning. Turner. **A.** 1072.
Nose and Throat Operations. Boyle. **A.**, 1557.
Spinal Applications. Savariaud. **A.**, 843.
Sub-Cutaneous. Gwathmey and Bliss. **A.**, 1467.
**Aneurism**, Excision. Moncany and Legendre. **A.**, 874.
**Anthrax**. **C.**, 143, 438, 274.
**Antigangrenous Serotherapy.** Vincent and Stodel. **R.**, 835.
**Antiseptics**. **E.**, 405, 889.
Perkins. **A.**, 1520.
Action. Taylor. **A.**, 830.
Toxicity. Taylor and Austin. **A.**, 832.
**Anti-Tetanic Serum**. **C.**, 273.
**Appendicitis**. **E.**, 240.
**Area of Advance,** Problems. 1252.
**Army Appropriation Bill**. **E.**, 424.
**Arsenobenzol**. **C.**, 140.
**Arthritis.** Rigaux and Gate. **A.**, 631.
Willems. **A.**, 860.
Septic. Neve. **A.**, 870.
Suppurating. Tridon. **A.**, 871.
**Arthrotomy.** Willems, **A.**, 860.
**Artificial Limbs**. Little, **A.**, 87.
**Asepsis**. **E.**, 405.
**Asthenia.** Laignel-Lavastine. **A.**, 44.
**Aviators,** Ear Disorders. Castex. **A.**, 1163.
Test. Robertson. **A.** 1120.

**Bacillus**, Influenza. Taylor and Austin. **A.**, 830.
Tubercle. A. E.F. **R.**, 988.
**Bacillus Welchii.** Stoddard. **A.**, 1517.
Taylor and Austin. **A.**, 830.
**Bacteria**. Weaver. **A.**, 1507.
Natural Defense Against. **C.**, 242.
**Bacteriological** Diagnosis. **C.** 442.
**Bacteriology**, Wound. Beebe. **A.**, 1023.
**Bladder**, Wounds. **I. S. C.**, 61.
Wounds. Head and Riddoch. **A.**, 71.
Wounds. Tanton. **A.**, 80.
Wounds. Tanton. **A.** , 235.
**Blood** Grouping. Shawan. **A.**. 359.
Pressure, 773.
Pressure. **E.**, 896.
Transfusion. **I. S. C. A.**, 58.
Transfusion. Harrison. **A.**, 216.
Transfusion. Robertson. **A.**, 68.
Transfusion. **R.**, 776.
Transfusion. Primrose. **A.**, 1069.
Transfusion Sets. **E.**, 907.
Vessel Suturing. Horsley. **A.** 218.
**Boils**, Nile. Crowe. **A.**, 1541.
**Bone**. See *Osteosynthesis*, 64.
Closure of Cavities. Sargent. **A.**, 1490.

**Bone**. Grafting. BANCROFT. A., 382.
Grafts. MILLER. A., 1494.
Graft. THURLOW and MACKLIN. A., 381.
Madderized. THURLOW and MACKLIN. A., 381.
Substance, Loss. MAYET. R., 847.
Surgery. BANCROFT. A., 382.
Surgery of Extremities. THOMPSON. A., 1492.
**Botulism.** DICKSON. A., 1422.
**Boveri's** Reaction. A., 646.
**Brain**. Wounds. E., 422.
Wounds. CUSHING. A., 69.
Wounds. MAIRET and PIERON. A., 1454.
**Bronchitis**, C., 696.
MACDONALD, RITCHIE and FOX. A., 1413.
MAUDE. A., 600.
VIOLLE. A., 636.
Bleeding. VIOLLE. A., 636.
Spirochetosis. VIOLLE. A., 636.
**Bronchopneumonia**. COLE and MAC CULLUM. A., 616.
MAC CALLUM. A., 623.
Measles. LUCKE. A., 632.
**Bubonic Plague**. SYMMERS. A., 1398.

**Cancers**. BEHARD. S., 845.
FORGUE. A., 844.
**Cardiac Tests**. LIAN. A., 1439.
**Cecostomy**. TAYLOR. A., 215.
**Cellulose Tissue**. C., 922.
**Cerebro-Spinal Fever,** Prevention. GLOVER. A., 1405.
SHORT. A., 1403.
**Chest**, Gun Shot Wounds. BRADFORD. R., 10.
Roentgen Study. FAVIS. A., 225.
Surgery. LE FORT. A., 852.
Surgery. LOCKWOOD. R., 6.
Surgery. SOLTAU. R., 1.
Surgery. TUFFIER. R., 16.
Surgery. DE LA VILLEON. A., 851.
Wounds. BLAIR and SHATTUCK. A., 1050.
Wounds. DEAN. A., 368.
Wounds. DOBSON. A., 76.
Wounds. E., 116.
**Chief Surgeon's Office**. E., 425.
**Chinese Laborers**. C., 143.
**Chloramine-T Solution**. AUSTIN and TAYLOR. A., 833.
**Chlorine**. C., 283.
**Colds**. VALENTINE. A., 613.
E., 666.
**Colon**, Resection. TAYLOR. A., 215.
**Conference** on Surgery in Battle Areas. CRILE, TUFFIER, BOWLBY, GASK, RICHARDS, SOLTAU, LOCKWOOD, BETZ, DEHELLY. R., 293.
**Contagious Disease**. CAPPS. A., 612,
**Convalescent Camps**. ASHFORD. R., 350.
NEEF. R., 757.
E., 419.
Depots. DALRYMPLE. R., 746.
**Convoys**. TARNOWSKY. A., 1500.
**Convulsions**. LAIGNEL-LAVASTINE. A., 44.
**Cough**, Chronic. R., 971.
**Cultures**, Enteric Cases. C., 444.

**"D" Patients**. C., 136.
**Deafness.** HAUTANT. R., 177.
JOBSON. A., 101.
**Decalogue for Disease Defense**. C., 693.
**Dengue**, 100 Cases. LANE. A., 1416.
**Dentistry**. See *Facio-Maxillary Surgery*. 1558-1578.
**Dermatology in B. E. F.** KNOWLES. A., 396.
**Diagnosis**, Bacteriological. E., 442.
Meningitis. E., 704.
Evacuation. E., 689.
**Diaphragm**. DE LA VILLEON. R., 24.
**Diaphyseal Fractures**. DELMAS. A., 380.
**Diarrhea**. C., 278.
**Dichloramin-T**. LEE and TURNER. A., 1150.
REIMANN and MAGOUN. A., 79.
**Dichlor-Ethyle-Sulphide**. (MUSTARD GAS). C., 283.
**Diet**, U. S. Camp. MURLIN. A., 1165.
**Dietitians**. C., 275.
**Diphtheria**. C., 1190,
Carriers. C., 131.
Gassed Cases. C., 452.
**Disabled**, Sailors and Soldiers. A., 90.
**Disinfection**, Method of. A., 1107.
**Donors**. 780.
**Dysentery**. C., 440.
COWAN and MILLIER. A., 1425.
Amebic. LAMBERT. A., 1111.
Germany. C., 443.
Serum Treatment. A., 1426.
Treatment of. LAWSON. A., 1427.

**Ear Affections**. HAUTANT. R., 176.
**Ear**, Deafness. JOBSON. 101.
Diseases of. O'MALLEY. A., 1556.
Disorders among Aviators. CASTEX. A., 1163.
Influence of Altitude. LEWIS. A., 1163.
**Eczema Marginatum**. C., 143.
**Elbow**, Wounds. MARTIN. A., 1064.

**Empyema.** Beals, Zimmerman and Barlow. A., 1399.
Brooks and Russell. A., 1094.
Camp Doniphan. Ingraham, Roddy and Aronson. A., 1400.
McKenna. A., 647.
Miller and Lusk. A., 648.
Tuffier. A., 1076.
Thorax. Lilienthal. A., 1480.
**Emulsions**, Microbic. Broughton-Alcock. A., 393.
**Encephalitis.** Vaidya. A., 1142.
Epidemic. Hall. A., 1105.
**Endocarditis.** Karsner. A., 1435.
Chronic. Warfield. A., 1123.
**Epidermitis**, Microbian. Gougerot. A., 1539.
**Epilepsy**, Jacksonian. Meige and Benisty. A., 391.
**Erysipelas**, Facial. Avata and Woodyatt. A., 1092.
**Ethyl-Chloride Ampoules.** Barlow. A., 1144.
**Evacuation**, Records. C., 907.
Wounded. C., 689.
Wounded. Ensor. A., 189.
Wounded. James. R., 199.
Wounded " D " Patients. C., 276.
**Eyeball**, Convergence. Euziere and Merle. A., 1136.
Lacerations. C., 453.
**Eye**, Conditions, Malta 1916-17. Maxwell. A., 1549.
Inspection. Derby. A., 1550.
Effect of Altitude. Wilmer and Berens. A., 1160.
Effects of Mustard Gas. Derby. A., 399.
Mustard Gas Effects. E., 451.
Wounds. E., 245.
Wounds. Wilson. A., 102.

**Facio-Maxillary Surgery.** Nodine and Kazarjam. R., 1370.
A., 1558-1578.
**Feces**, Disposal. Coburn. R., 339.
Gressinger. R., 333.
Joscelyne. R., 327.
Monod. R., 324.
**Femoral Artery**, Surgery. Nicoll. A., 1495.
**Femur**, Fractures. E., 258.
Infected Hematoma. Bernard. A., 869.
Resections. Leriche. A., 868.
**Fever**, Cerebro-Spinal. Short. A., 1403.
Scarlet. Warrack. A., 1411.
Three Day. Joly and Baril. A., 605.
Trench. C., 439; A., 96, 247; E., 897.
Trench. Rudolph. A., 1418.
**Fever.** Typhoid. Hulton-Frankel. A., 1510.
Typhoid. C., 440.
Typhoid, Diagnosis. Tribondeau. A., 1509.
Typhus. C., 439.
**Filariasis.** Australian Troops. Rimmer. 1437.
**Fleas.** C., 453.
**Flies**, Destruction of. A., 1164.
**Fluoroscopic Examination.** Borzell. A., 1545.
**Fly Prevention.** Coburn. R., 339.
Gressinger. R., 333.
Joscelyne. R., 327.
Monod. R., 324.
C., 139.
**Food.** C., 444.
**Foot Wounds.** A., 62.
**Foreign Bodies.** Le Fort. A., 217.
Picknell. A., 857.
Extraction. Didier. A., 850.
Extraction. Marion. A., 372.
Extraction. De la Villeon. A., 372; R., 24; A., 851.
Migration. Achard and Binet. A., 840.
**Formalin.** Guillaume-Louis and Rousseau. A., 834.
**Fractures.** A., 64.
C., 425.
Baer. A., 82.
Thevenot and Tuffier. A., 839.
Compound. W. L. and C. P. Brown. A., 1053.
Diaphyseal. Delmas. A., 380.
Extremities. Frost. A., 862.
Long Bones. Leriche and Policard. A., 1063.
Thigh. Blacke. R., 150.
Thigh. Heitz-Boyer. R., 157.
Thigh. Leriche. R., 153.
Thigh. Sinclair. R., 145.
Treatment. Nifong. A., 1067.
Warfare. Lane. A., 1060.

**Gangrene.** Ombredanne. A., 361.
**Garbage.** C., 446.
**Gas Gangrene.** Christopher. A., 1464.
Hartley. A., 1147.
Ivens. A., 1148.
Nevin. A., 1514.
Pathology. McIntosh and McNee. A., 1519.
**Gas**, Mustard. C., 283.
Mustard. Warthin. A., 1441.
Mustard. Effects on Eyes. C., 451.

**Gas**, Mustard, Effects on the Eyes. DERBY, A., 399.
Pains. EMGE. A., 1101.
Phosgene. C., 283.
Poisoning. C., 449, 911.
Poisoning. UNDERHILL, GOLDSCHMIDT and WILSON. A., 1525.
Poisoning. Prevention. C., 281.
**Gases**. Arsenic. C., 450.
A., 94.
**Gas School**. A. E. F. E., 903.
**Gauze**, Salvaging and Washing. C., 924.
**G. H. Q. General Orders**. Medical Service in the Am. E. F. 927, 1217.
**Gonorrhea**, Complement Fixation Test. BOWMAN and TAYLOR. A., 1151.
**Grippe**. See *Influenza*, VIOLLE. A., 598.
Prophylaxis. CHAUFFARD, NETTER, VINCENT, ACHARD and BEZANCON. A., 1098.
Treatment of. LYON. A., 1113.
**Gum-Salt Solution**. 784.
**Gun-Shot Fractures of the Extremities**. E., 1179.
**Gutta-Percha**. **Tissue**. Substitute. C., 922.

**Hand**, Infection. BEYE. A., 854.
**Head**, Surgery. FRAZIER. A., 364.
Surgery. ROBERTS. A., 1473.
**Headaches**. Sinus Origin. IBBOTSON. A., 1553.
**Heart**, Disordered Action of. C., 134.
Effect of Trench Fever. CREAM and BARTON. A., 96.
Functional Disorder. MACILUAINE. A., 224.
Irritable. WARFIELD and SMITH. A., 1438.
Surgery. CONSTANTINE and VIGOT. A., 374.
Tuberculosis. R., 981.
Wound. BOND, PHILLIPS and JEVONS, A., 1049.
**Hemorrhages**. R., 770; E., 895.
PENFIELD. A., 1521.
RICHET and SAINT-GIRONS. A., 1071.
Secondary. NEUHOF and ST. JOHN. A., 1458.
Urinary Symptoms. PETERS and STEVENS. A., 1528.
**Hernia**, Strangulated. C., 142.
**Hip-joint**, Resection. ALQUIER and TANTON. A., 868.
Resection. LERICHE. A., 868.
Resection. POTHERAT. A., 866.
**Hospitals**, Cantonment, MERRILL. A., 105.
Casualty Clearing Stations, WOOLER. A., 1075.
**Hospitals**. Evacuation. JONES. A., 1498.
French. C., 125.
Italian. BAER and WILSON. A., 218.
Organization. R., 294.
Paris. E., 1177.
Paris. BOULANGER. R., 1037.
**Hospital Trains**. C., 277.
**Hygiene**, Personal. E., 413.
**Hypochlorite**, Solution. Action on Wounds. A., 360.
Solutions. AUSTIN and TAYLOR. A., 833.
**Hysteria**. Base Hospital. SYMNS. A., 1446.
Military Hospital. TROTTER. A., 1145.
Treatment of. WOLFSOHN. A., 1449.

**Icterus**, in Roumania. CANTACUZENE, A., 1430.
**Infections**, E., 668.
MARTIN. A., 1463.
Hand. BEYE. A., 854.
Pulmonary. RIGAUX and GATE. A., 631.
Respiratory. BEVERIDGE. R., 300.
Respiratory. ZINSSER. R., 316.
Respiratory. BOREL. R., 306.
Respiratory. EMERSON. R., 311.
**Infections** Diseases. C., 1207.
**Influenza**. A., 609, 595, 596.
C., 137, 278, 690, 1203.
E., 666, 885.
AVERILL, GRIFFITHS. A., 603.
MAUDE. A., 600.
ROBERTSON. A., 228.
SYMMERS. A., 1398.
SYMONDS. A., 1392.
VIOLLE. A., 598.
WIRGMAN. A., 602.
Bacillus. A., 607.
Bacillus. BROWN and ORCUTT. A., 1506.
Bacteriological Study. BARBIE and LECLERC. A., 610.
Base Hospital. BRADBURY and KRUMBHAAR. A., 1383.
B. E. F. A., 1385.
B. E. F. France. MARTIN. A., 600.
Camp Sherman. FRIEDLANDER, McCORD, SLADEN and WHEELER. A., 1390.
Control. E., 887.
Navy Hospital. HEWLETT and ALBERTY. A., 604.
Open Air Treatment. BROOKS. A., 1391.
Prophylactic. VINCENT and LOCHON. A., 608.
Serums and Vaccines. E., 1391.
Use of Human Serum. McGUIRE and REDDEN. A., 1395.
**Influenzal** Sinus Disease. ROBERTSON. A., 228.

**Interallied Surgical Conference**. 4th. Session. A., 57.

**Jaundice**. Neill. A., 1428.
Infectious. C., 135, 141.
**Jaw**. Fractures. Roberts. A., 1473.
Injuries. Bennett. A., 90.
Wounds. Pickerell. A., 857.
**Joint**, Injuries. Rouvillois and Guillaume Louis. A., 859.
Wounds. C., 425.

**Kidneys**. Fitz. A., 227.
Resection. Simonin. A., 1156.
**Kineplastic** Amputation. Gaudeans. A., 378.
Amputation. Putti. A., 376.
**Knee-Joint**, Arthrotomies. Mouchet and Pamart. A., 871.
Resection. Tridon. A., 871.
Wounds. Charles. A., 374.
Wounds. Cumston. A., 872.
Wounds. Metcalf. A., 1496.
Wounds. Speed. A., 83.

**Laboratory**, Base. Boney, Crossman, Boulenger. A., 382.
Technic. Morrill. A., 1505.
Technic. Tribondeau. A.. 1502.
**Larynx**. Wounds. Harmer. A., 100.
**Leg**, Fracture. Sneyd. A., 86.
Surgery. Phocas. A., 875.
**Lesions**, Peripheral Nerve. McDonald. A., 234.
**Lice**. Ensor. R., 170.
**Lice-Borne** Diseases. Hunter. A., 1107.
**Limbs**, Artificial. See : *Artificial Limbs.*
**Lungs**, Infection. Wilson. R., 556.
Operative Results. Duval. R., 21.
Pneumonic. Weiss, Kolmer, Steinfield. A., 634.
Resection. Gregoire. A., 370. 1056.
Surgery. Marion. A., 372.
Surgery. Duval. R., 21.
Wounds, Eggers. A., 77.
X. Ray. de la Villeon. A., 372.
**Lyster** Water Sterilizing Bag. C., 276.

**Malaria**. Pepin. A., 1117.
Cantonments. LePrince. A., 1112.
Problems. Hoffmann. A. 1436.
Treatment Falconer and Anderson. A., 1102.
**Mange**. C., 142.
**Masks**. Contagious diseases. C., 452.
Contagious diseases. Capps. A., 612.
**Measles**. Levy. R., 560.
Cole. A., 622.
C., 1208.
**Measles**. MacCallum. A., 616.
Macdonald, Ritchie and Fox. A., 1413.
Pratt. A., 615.
Bronchopneumonia. Lucke. A., 632.
Diagnosis. Warrack. A., 1411.
**Medical Corps**. E., 654.
**Medical Department**. C, 126; E, 659, 880
Funds. C., 277.
**Médical Education**, C., 429.
id. **Officers**. C., 248.
id. **Organisation**. Italian Army. A., 1453.
id. **Reserve Corps**. C., 267; E., 879.
id. **Service**. E., 876.
**Meningitis**. E., 438.
C., 690.
A., 646, 221.
Fitzgerald. A., 1103.
Flexner. A., 641.
Rosanoff. A., 1406.
Diagnosis. C., 704.
Epidemic. Cohen and Fle ming. A. 1508.
Epidemic. Mathers and Herrold. A. 638.
Cerebro-Spinal. Pacchioni. A , 639.
Pyocyanic. Abadie and Laroche. A. 642.
**Meningococcus**. Bloch and Hebert. A., 642.
Gordon. A., 644.
Carriers. Mathers and Herrold. A., 638.
Effect of Acids. Shaw-Mackensie. A., 645.
Serum. A., 438.
**Mosquitoes**, Destruction of. A., 1164.
**Mumps**, Camp Wheeler. Radin. A., 1118.
**Muscle**. E., 418
**Museum**, Material. C., 429.
**Mustard Gas**. See *Gas, Mustard.*
**Myasthenia**, Neurocirculatory. Mac Farlane. A., 1440.
**Myoplastic** Operations. Letarget. A., 865.

**Nephritis**. Dyke. A., 1421.
McLean. A., 1115.
Early Stages. Strathy. A., 1420.
**Nerve** Growth. Cone. A., 1128.
Injuries. Thomas., 1351.
Injuries. Bibliography, 1362.
Isolation. Meuriot and Platon. A., 850.
Lesions. McDonald. A., 234.
Peripheral. Debaisieux. A., 1476.
Peripheral. Gibson. A., 1455.

**Nerve**. Peripheral. MacKenzie. A., 1138.
Peripheral. Repair by Neuroplasty. MacKenzie. A., 1138.
Peripheral Surgery. Stookey. A., 1140.
Peripheral. Venable. A., 1478.
Suture. Nageotte. A., 848.
Wounds. Stopford. A., 232.
Wounds. Tinel. A., 209.
C. of S. A., 1130.
**Neuro-Otologic Tests**. Carpenter. A., 1457.
**Neuroses**. E., 118.
Dupre. R., 31.
Henderson. A., 231.
Kennedy. R., 26.
Terhune. A., 99.
Diagnosis and Treatment. Laignel-Lavastine. R., 44.
Effect on Limbs. Roussy. R., 37.
Prevention. Rhein. R., 47.
Treatment. Salmon. R., 34.

**Oil**, Olive. Horsley. A., 218.
Sanitation. C., 454.
**Ophthalmology**, Military. McKee. A., 1158, 1548.
**Orthopedics**. C., 425.
**Osteosynthesis**. A., 64.
**Oto-Laryngeal Affections.** Hautant. A., 176.

**Paralysis**. Functional. Brow and Stewart. A., 388.
Pneumogastric. Vernet. A., 629.
Pseudo. Hunt. A., 390.
**Paratyphoid**. Hulton-Franke. 1510.
Agglutins. McAdam. A., 1511.
**Pelvis**. Tanton. A., 80.
Surgery. Churchman. A., 853.
Wounds. A., 61.
Wounds. Tanton. A., 235.
**Peritoneum**. Reimann and Magoun. A., 79.
**Petrol**, Dermatitis. Page. A., 238.
**Phosgene Gas**. See *Gas, Phosgene.*
**Physical Examination**. Bushnell. A., 653.
Trudeau. A., 650.
Wheaten. A., 650.
**Pigs**. C., 446.
**Pleura**. de la Villeon. E., 24.
**Pleurisy**. Treatment. Guthrie. A., 1104.
**Pneumonia.** C., 696, 690, 688, 1210.
E., 664, 885.
Cummins and Lynch. A., 619.
Dickson. A., 1395.
Hamburger and Mayers. A., 626.
Longcope. R., 566.
Pratt. A., 615.
**Pneumonia**. Small. A., 624.
Symmers. A., 1398.
Bacteriology. Dick. A., 625.
Base Hospital N° 53. Goodkind. A., 1393.
Blood Cultures. McClelland. A., 1397.
Catechism. E., 886.
Complications. R., 974.
Control. E., 887.
Contagion. Valentine. A., 613.
Etiology. MacCallum. A., 616.
Lobar. Kolmar and Steinfield. A., 614.
Naval Hospital. Deyer and Boles. A., 1396.
Prevention. Cole. A., 620.
Prophylaxis. Kolmer and Steinfield. A., 614.
Resistance Against. E., 669.
Treatment. Guthrie. A., 1104.
Urine. Quigley. A., 630.
Use of Human Serum. McGuire and Redden. A., 1395.
**Pneumococcic Infection**. Rigaux and Gate. A., 631.
**Pneumococcus Groups**. Cummings, Spruit and Lynch. A., 619.
**Posterior Thoraco-Pneumotomia** Petit de la Villeon. A., 372.
**Post-Mortem Findings**. Lucke. A., 632.
**Pseudarthroses**. I. S. C., 62.
**Psychosis**. See *Neuroses.*
**Pyodermia**. C. 289.
**Publications**. E., 414.

**Quinine**. Pepin. A., 1117.

**Rabies**. C., 143.
**Radial Nerve**. Paralysis. Ripert. A., 1191.
**Radiography**. Technic. Bougourd. A., 1547.
Technic. Fornario. A., 398.
**Radium**. Laborde. A., 838.
**Rank for Medical Officers**. E., 423.
**Reclus' Method**. Lapeyre. A., 841.
**Reconstruction**. Broca. A., 1090.
Agricultural Work. Bergonié. A., 1087.
Artificial Limbs. Galleazi and Lollini. A., 1090.
Physical. E., 900.
War Crippled. Gourdon. A., 1088.
Belgium. Melis. A., 1091.
**Rectum**, Wounds of. A., 61.
Tanton. A., 80, 235.
**Recuperation of Wounded**. Dalrymple. R., 746.
Laignel-Lavastine. A., 751.

**Research**, A. E. F. C., 429.
Society. E., 106.
Society Meetings. E., 658.
**Respiratory Diseases**. BEALS, ZIMMERMAN and BARLOW, 1399.
Infections. C., 436.
Infections. E., 660.
Infections. E., 407.
Infections. BEVERIDGE. R., 300.
Infections. CAPPS. R., 571.
Infections. VERNET. A., 629.
Infections. ZINSSER. R., 316.
Infections. American Expeditionary Forces. C., 678.
Infections. American Expeditionary Forces. EMERSON. R., 311.
Infections. French Army. BOREL. R., 306.
**Restoration of Wounded**. BILLINGS. R., 766.
**Rubella**. MACDONALD, RITCHIE and FOX. A., 1413.
Diagnosis. WARRACK. A., 1411.
**Russian Army**. HAZLETT. A., 1444.

**Salvage**. C., 419.
**Sanitation**. E., 412.
C., 454, 436.
MONOD. R., 324.
**Scabies**. KNOWLES. A., 1540.
In Military and Civil Life. KNOWLES. A., 237.
**Scarlet Fever**. Diagnosis. WARRACK. A., 1409.
**Scars**, Abdominal. Painful. QUAIN and EGGERS. A., 1487.
**Scopolamine**. LAPEYRE. A., 841.
**Sensation**, Absence of all. ROBERTS. A., 1451.
**Serum**, Anti-Endotoxin. GORDON. A., 644.
Intraspinal. ABADIE and LAROCHE. A., 642.
Intraspinal. HEBERT and BLOCH. A., 642.
Meningococcus. E., 438.
Serotherapy. VINCENT and STODEL. A., 835.
**Sheath**, India Rubber, MEURIOT and PLATON. A., 850.
**Shell-Shock**. E., 119, 244.
Effects on Eye. LACROIX. A., 1548.
**Shock**. E., 418.
EUZIERE and MERLE. A., 1136.
Blood Pressure in. E., 896.
Diagnosis. MOREAU and BENHAMOU. A., 1066.
Prognosis. MOREAU and BENHAMOU. A., 1066.
**Shock**. Remedy. C., 279.
Shell. DILLON. A., 1137.
Surgery. R., 785.
Surgical. SWEET. A., 358.
**Shock**, Traumatic. R., 770; E., 895.
Treatment. MOREAU and BENHAMOU. A., 1066.
"**Shock Teams**". C., 907.
**Shoulder**. Resection. LETARGET. A., 865.
Surgery. BERTEIN. A., 865.
**Skin Affections**. Soldiers. HAILPERIN. A., 1539.
Disease. KNOWLES. A., 237.
Disease. PAGE. A., 238.
Grafting. MASSON. A., 67.
Grafting. SHAWAN. A., 359.
**Skull Wounds**. BINET. A., 1127.
TERRIEN. A., 1159.
**Slide Method**. BROUGHTON-ALCOOK. A., 393.
**Solution**, Gum Salt. R., 784.
Hypochlorite. FIESSINGER and CLOGNE. R., 360.
Chloramine-T. AUSTIN and TAYLOR. R., 833.
Hypochlorite. AUSTIN and TAYLOR. R., 833.
**Sores**, Septic. CROWE. A., 1541.
**Spanish Flu**. See *Influenza*.
**Spine**. Injuries. HEAD and RIDDOCH. A., 71.
Surgery. FRASIER. A., 75.
**Spirochetosis**. BARBARY. A., 637.
Broncho-Pulmonary. VIOLLE. A., 636.
Pulmonary Hemorrhagic. BARBARY. A., 637.
**Sputum**. Laboratory Technic. ECKER. A., 1506.
**Sterilization of Wounds**. WRIGHT, FLEMING, COLEBROOK. A., 203.
**Streptococcic Wounds** DE ARRIC. A., 1143.
**Streptococcus**. E., 661.
RIGAUX and GATE. A., 631.
Epidemic. FOX and HAMBURGER. A. 628.
Groups. CUMMINGS, SPRUIT and LYNCH A., 619.
Haemolyticus relative to Pneumonia. LONGCOPE. R., 566.
Infections. WILSON. R., 556.
Infection. CAPPS. R., 571.
Infection following Measles. LEVY R., 560.
**Stumps**. DESFOSSES. A., 379.
LITTLE. R., 87.
**Surgery at the Base**. FINNEY, CRILE and BURNETT. R., 1281.
Battle Areas. E., 402.

**Surgery at the Base**. Facio-Maxillary. Nodine and Kazarjam. R., 1370.
Intrathoracic. R., 1000.
Plastic. Mayet. A., 847.
Thoracic Drainage. Meyer. A., 1065.
War. Hartwell and Butler. A., 1058.
**Surgical Operations**. C., 124.
**Suture**. Baer. A., 82.
R., 593.
Ehrenpreis. A., 1461.
Horsley. A., 218.
Lemaitre. A., 206; R., 790.
Sencert. A., 840.
Thevenot and Tuffier. A., 839.
Nerves. Nageotte. A., 848.
Wounds. E., 888.
**Sweating**. Head and Riddoch. A., 71.
**Syndrome**. Cohn. R., 761.
**Syphilis**. Incubation Period. Jambon and Tzanck. A., 1154.
**Syringomyelia**. Meuriot and Lhermitte. A., 1452.

**Tetanus**. Antoine. A., 229.
Bruce. 724.
Gessner. A., 1471.
Localized. Chauvin. A., 230.
**Thigh**, Fractures. Heitz-Boyer. R., 157.
Leriche. R., 153.
Sinclair. R. 145.
**Thoracic Drainage**. Meyer. A., 1065.
**Thoracic Injuries**. Lilienthal. 1485.
**Three Day Fever**. C., 278.
Joly and Baril. A., 605.
**Throat**, Vincent's Angina. Greeley. A., 98.
**Tissue**, Cellulose. 922.
Gutta Percha. 922.
Inflammation Connective. Knowles. A., 396.
Necrotic and Normal. Austin and Taylor. R., 833.
**Tonsil**, Infections. Nichols and Bryon. A., 1555.
Vincent's Angina. Greeley. A,. 98.
**Trachoma**. C., 143.
Cary. A., 1552.
**Transfusion Sets**. C., 907
**Transfusions**. Penfield. A., 1521.
**Transportation**. C., 687.
Wounded. C., 264.
Wounded. Ensor. R., 189.
Wounded. Gallie. R., 184.
Wounded. Poe. R., 191.
Wounded. Proust. R.,196.
Wounded. Thompson. R., 194.
Wounded in Trenches. Graves. A., 362.
**Trauma**, War. Behard. A., 845.
**Traumatic Shock**. Cannon. R., 1367.
**Tremors**. Meige and Benisty. A., 392.
**Trench Feet**. E., 902.
I. S. C. A., 59.
Treatment. Ashford. R., 707.
Pathology. Cottet. R., 707.
Prevention. Fuhr. R., 712.
**Trench Fever**. E., 247, 439.
Cream and Barton. A., 96.
Rudolph. A., 1418.
Sweet. A., 1121.
Investigations. E., 897.
**Tuberculosis** E., 891, 1175.
C., 453, 705, 696, 125.
R., 972.
Trudeau. A., 650.
A. R. C. in France. White. A., 1124.
Army Problem. Brown and Pratt. A., 1096.
Autopsy. R., 993.
Diagnosis. R., 971.
Early Diagnosis. R., 998.
Examination Methods. Kahn. A., 1407.
Heart Conditions. R., 981.
Koch's Bacillus. Laroche and Virmeaux. A., 1503.
Lesions. R., 991.
Physical Examination. Bushnell. A., 653.
Physical Examination. Wheaton. A., 650.
Pseudo. Barbary. A., 637.
Pulmonary. Army. R., 990.
Pulmonary. Diagnosis. R. 985, 989.
Treatment. Bushnell. A., 651.
Kidneys. C., 141.
**Tumors**. Vitrac. A., 845.
**Typhoid Fever**. Hulton-Frankel. A., 1510.
Bacteriological Diagnosis. Tribondeau. A., 1509.
**Typhoid Spine**. Osler. A., 1116.
**Typhus Fever**. E., 439.
Craig and Hamilton. A., 1100.
Eastern Europe. Baehr and Plotz., 1419.

**United States**. Army Changes. E., 654.
**Urea**. Index. Fitz. A., 227.
**Uremia**. Reynes. A., 1466.
**Urine**. Garnier and Reilly. A., 1432.
MacLean. A., 1115.
Watson. A., 1504.
in Pneumonia. Quigley. A., 630.
**Urology**. Military. E., 252.
Post-Gonorrheal Conditions. Smith. A., 1532.
Resection of Penis. de Sard. A., 1530.
**Urticaria**. C., 138.

**Vaccines.** Wright. A., 1513.
**Venereal Diseases.** E., 1174.
Gerard. A., 1525.
Peacock. A., 1537.
Army. Thompson. A., 1157.
Gonorrhea. Chapin. A., 1522.
Public Health Problem. Irvine. A., 1533.
Treatment. Qualls. A., 1155.
Problem. E., 1180.
Problem. U. S. Army. Pusey. A., 1535.
Prophylaxis. C., 283.
**Vincent's Angina.** Barker and Miller. A., 1093.
France. Bouty. A., 1409.
Gallaher. A., 1409.
Greeley. A., 98.
Taylor and MacKinstry. A. 1408.

**"War Medicine".** E., 108, 250.
**War**, Medical Methods. Fotheringham. A., 1441.
Neuroses. See *Neuroses*.
Wounds. Cure. Bergonié. A., 1087.
Wounds. Treatment. C., 121.
Wounds. Treatment. Kouindjy. A., 1489.
**Wassermann Reaction.** Browning and Kennaway. A., 1523.
Reaction. d'Este Emery. A., 1152.
Reaction. Thibierge. A., 1538.
**Wastage.** E., 260.
British Army. Bartchael. R., 165.
Disease. C., 261.
Disease. A. E. F. Emerson. R., 178.
Disease. Ensor. R., 170.
Lice. Ensor. R., 170.
Prevention. Brown. R., 173.
Problem. Cowie. R., 175.
**Water Borne Disease.** Boney, Crossman, Boulenger. A., 382.
Orders Regarding Purification. C. 435.
**Weil-Félix Reaction.** Craig and Hamilton. A., 1100.
**Wound** Bacteriology. Beebe. A., 1023.
Infection. Ombredanne. A., 361.
**Wounded.** C., 919.
Recuperation. Dalrymple. R., 746.
Recuperation. Laignel-Lavastine. R., 751.
**Wounded**, Slightly. C., 125; R., 299.
**Wounds.** E., 240.
Lemaitre. R., 790.
Stoddard. A., 1517.
Brain. E., 422.
Brain. Cushing. A., 69.
Brain. Mairet and Pieron. A., 1454.
Chest. Blair and Shattuck. A., 1050.
Chest. Bradford. 10.
Chest. Dean. A., 368.
Chest. Soltau. 1.
Chest. Tuffier. R., 16.
Contamination and Infection. Crile. A., 65.
Elbow. Martin. A., 1064.
Encephalic. Moulonguet and Legrain. A., 209, 846.
Genital Organs. Cumston. A., 1529.
Gunshot. Dobson. A., 366.
Gunshot. Upcott. A., 1073.
Heart. Bond, Phillips and Jevons. A., 1049.
Heart. Constantine and Vigot. A., 374.
Infected. Crile. A., 65; R., 587.
Jaw. Pickerill. A., 857.
Knee-Joint. Charles. A., 374.
Knee-Joint. Neve. A., 870.
Nerve. Stopford. A., 232.
Nerve. Tinel. A., 209.
Pelvic. Tanton. A., 235.
Perforating. Drumond and Fraser. A., 1055.
Problems. R., 297.
Skull. Lemere. A., 1159.
Sterilization. Wright, Fleming, Colebrook. A.. 203.
Streptococcic. de Arric. A., 1143.
Suture. E., 888.
Suture. Lemaitre. R., 206, 790.
Thoracic. R., 982.

**X-Ray.** Examination of Lungs. Mantoux and Maingot. A., 1544.
Extraction of Foreign Bodies from Lung. Petit de la Villeon. A., 372.
Localization. McGrigor. A., 1542.
War Surgery. Bettman. A., 1546.

### INDEX OF AUTHORS

**Abadie, MM. J.**, p. 642.
**Achard, MM. Ch.**, pp. 840, 1098.
**Alberty, W. M.**, p. 604.
**Allison, R. J.**, p. 984.
**Alquier, MM.**, p. 868.
**Anderson, A. G.**, p. 1102.
**Antoine, Edward**, p. 229.
**Aronson, J. D.**, p. 1400.
**Arric, Le Fevre de**, p. 1143.
**Ashford, Bailey K.**, pp. 657, 350, 717.
**Austin, J. Harold**, pp. 830, 832, 833.
**Avata, Anthony A.**, p. 1092.
**Averill, C.**, p. 603.

**Baehr, G.**, p. 1419.
**Baer, William S.**, pp. 82, 218.
**Bancroft, Frederic W.**, p. 382.
**Barbary, F.**, p. 637.
**Baril**, p. 605.
**Barker, L. F.**, p. 1093.
**Barlow., F. C.**, p. 1144.
**Barney**, p. 587.
**Barton, B. H.**, p. 96.
**Beals, L. S.**, p. 1399.
**Beebe, Theodoro C.**, p. 1023.
**Behard, L.**, p. 845.
**Belanger, Ad.**, p. 1569.
**Benhamou**, p. 1066.
**Bennett, Norman G.**, p. 90.
**Berens**, p. 1160.
**Bergonié, J.**, p. 1087.
**Bernard, J.**, p. 869.
**Bernard, Léon**, p. 983.
**Bertein, M.**, p. 865.
**Bettman, Ralph B.**, p. 1546.
**Betz**, p. 293.
**Beveridge, W. W. O.**, p. 300.
**Beye, Howard L.**, p. 854.
**Bezançon**, p. 1098.
**Billings**, p. 766.
**Billington, Wm.**, p. 1559.
**Binet, M. L.**, pp. 840, 1127.
**Bjerving, C.**, p. 980.
**Blair, R. B.**, p. 1050.
**Blake, Joseph**, p. 150.
**Bliss, Sidney W.**, p. 1467.
**Bloch, Marcel**, p. 642.
**Bloch, René**, p. 829.
**Boles, M. C.**, p. 1396.
**Bond., C. J.**, p. 1049.
**Boney, T. K.**, p. 382.
**Borel**, pp. 321, 306.
**Borzell, Francis G.**, p. 1545.
**Bougourd**, p. 1547.
**Boulanger, T. D.**, pp. 116, 1037.
**Boulenger, C. L.**, p. 382.
**Bound, Harold**, p. 1559.
**Bouty, R. J. C.**, p. 1409.
**Bowlby, Anthony**, pp. 293, 587.
**Bowman, F. B.**, p. 1151.
**Boyle, Edmund G.**, p. 1557.
**Bradbury, S.**, p. 1383.
**Bradford, Sir John Rose**, pp. 10, 576.
**Broca, A.**, p. 1090.
**Broca, M.**, p. 1052.
**Brock**, p. 587.
**Brooks, Harlow**, p. 1094.
**Brooks, Wm. A.**, p. 1391.
**Broughton-Alcock, W.** p. 393.
**Brown, C.**, p. 173.
**Brown, C. P.**, p. 1053.
**Brown, James Howard**, p. 1596.
**Brown, Lawrason**, p. 1096.
**Brown, T. G.**, p. 388.
**Brown, W. L.**, p. 1053.
**Browning, C. H.**, p. 1523.
**Bruce, Sir David**, pp. 722, 724, 744.
**Bruns, E. H.**, p. 995.
**Bryan, J. H.** p. 1555.
**Bureau, Marcel**, p. 1054.
**Burnett, T. W.** p. 28.
**Burtchaell**, pp. 587, 323, 255, 165.
**Bushnell, G. E.**, pp. 653, 651.
**Butler, E.**, p. 1558.
**Butler, Ethan F.**, p. 1058.

**Cabot, Richard C.**, p. 978.
**Cannon, W. B.**, p. 1367.

**Cantacuzene**, Z., p. 1430.
**Capps, Joseph** A., pp. 571, 612.
**Carlson**, pp. 585, 744.
**Carpenter**, E. R., p. 1457.
**Cary**, E. H., p. 1552.
**Castellani**, **Aldo**, p. 986.
**Castex**, **André**, p. 1163.
**Cattel**, p. 579.
**Cattell**, **Henry** W., p. 993.
**Cecil**, **Russell** L., p. 1094.
**Chaput**, H., p. 842.
**Charles**, **Richard**, p. 374.
**Chauffard**, p. 1098.
**Chauvin**, E., p. 230.
**Chief Surgeon**, pp. 124, 114.
**Christopher, Frederick**, p. 1464.
**Churchman, John W**, p. 853.
**Clogne**, **René**, p. 360.
**Coburn**, p. 339.
**Cohen, M. B.**, p. 1508.
**Cohn, Alfred E.**, pp. 761, 981.
**Cole**, **Rufus**, p. 620.
**Colebrook**, L., p. 203.
**Cloyer, Claud G.**, p. 1572.
**Cone, Sydney** M., p. 1128.
**Constantine**, H., p. 374.
**Constantini**, p. 1084.
**Cottet**, **J.**, p. 707.
**Cowan, John**, p. 1425.
**Cowie**, p. 175.
**Craig, C. M.**, p. 1100.
**Cream, T. J.**, p. 96.
**Crile, George W.**, pp. 65, 293, 587.
**Crossman, L. G.**, p. 382.
**Crowe, H. Warren**, p. 1541.
**Cummings, J. G.**, p. 619.
**Cummins**, pp. 348, 723, 745.
**Cumston, Charles G.**, pp. 872, 1529.
**Cunningham, W. F.**, p. 208.
**Cushing, Harvey**, p. 69.

**Dalrymple**, p. 746.
**Davis, E. L.**, p. 225.
**Dean, C.**, p. 368.
**Deaver, John B.**, p. 1084.
**Debaisieux, Georges**, p. 1476.
**Dehelly**, p. 273.
**Delagenière, H.**, p. 1130.
**Delbet**, **Pierre**, p. 1145.
**Delmas, J.**, p. 381.
**Delorme**, p. 1131.
**Derby, George S.**, pp. 399, 1550.
**Descomps**, P., p. 1136.
**Desfosses, P.**, p. 379.
**Desjardins, A. N.**, p. 994.
**Desplas, M.**, p. 1086.
**Deyer, F. J.**, p. 1396.
**Dick, George F.**, p. 625.
**Dickson, Elliot.**, p. 1395.
**Dickson, Ernest C.**, p. 1420.
**Didier, Robert.** p. 850.
**Dillon, F.**, p. 1137.
**Dobson, J. F.**, pp. 366, 76.
**Drummond, Hamilton**, p. 1055.
**Dujarier**, p. 1133.
**Dupré**, p. 31.
**Duval, Pierre**, p. 21.
**Dyke, L. C.**, p. 1421.

**Ecker, E. E.**, p. 1506.
**Eggers, C.**, pp. 77, 1487.
**Ehrenpreis**, p. 1461.
**Elder**, p. 587.
**Emerson, Haven**, pp. 178, 311, 323, 583.
**Emery, W. d'Este**, p. 1152.
**Emge, L. A.** p. 1101.
**Ensor, H.**, pp. 17, 189.
**Ernst, E. C.**, p. 994.
**Euziere, J.**, p. 1136.

**Fairley, N. Hamilton**, p. 1100.
**Falconer, A. W.**, p. 1102.
**Fiessinger, Noël**, p. 360.
**Finney, J. M. T.**, p. 657.
**Fitz, Reginald, M.R.C.**, pp. 227, 1313.
**Fitzgerald, J. G.**, p. 1103.
**Flandin**, p. 982.
**Fleming, A.**, p. 203.
**Fleming, J.S.**, p. 1508.
**Flexner, Simon**, p. 641.
**Forgue, M. Em.**, pp. 844, 1133.
**Fornario**, p.. 398
**Fotheringham, J. T.**, p. 1443.
**Fox, Herbert**, p. 628.
**Fox, J. C.**, p. 1413.
**Francine, A. P.**, p. 984.
**Fraser, John**, p. 1055.
**Frazier, Charles H.**, p. 75.
**Friedlander, A.**, p. 1390.
**Frost, Harold**, p. 862.
**Fuhr, R. S. H.**, p. 712.

**Galippe, M. V.**, p. 1146.
**Gallaher, T. J.**, p. 1409.
**Galleazi, R.**, p. 1009.
**Gallie, F. S.**, p. 184.
**Garnier**, p, 1432.
**Garvin**, p. 974.
**Gask**, pp. 293, 587.
**Gate, M. J.**, p. 631.
**Gaudeans, Vincent**, p. 378.
**Gérard, P.**, p. 1525.
**Gernez**, pp. 1133, 1569.
**Gessner, Hermann B.**, p. 1471.
**Gibson, Alexander**, p. 1455.
**Glennan, James D.**, p. 557.
**Glomsett, D. J.**, p. 993.

**Glover, J. A**, p. 1405.
**Goldschmidt, S.**, p. 1525.
**Goldthwait, J.**, p. 723.
**Goodkind, M. L.**, p. 1393.
**Gordon, M. H.**, p. 644.
**Gorgas, W. C.**, p. 654.
**Gougerot, H.**, p. 1539.
**Gourdon, J.**, p. 1088.
**Graham**, p. 587.
**Graves, B.**, R. A. M. C., p. 362.
**Grégoire, M.**, p. 1056.
**Grégoire, Raymond**, p. 370.
**Gressinger, J. W.**, p. 333.
**Griffiths, J.**, p. 603.
**Gruley, Horace**, p. 98.
**Guillaume-Louis**, pp. 834, 859.
**Guthrie, A. Cowan**, p. 1104.
**Gwathmey. J-T.**, pp. 1057-1467.

**Hailperin, C. J.**, p. 1539.
**Hall, Arthur**, p. 1105.
**Halstead, T. H.**, p. 116.
**Hamburger, Walter W.**, pp. 626, 628.
**Harmer, W. Douglas**, p. 100.
**Harris Seale**, pp. 109, 582.
**Harrison, Benjamin I.**, pp. 216, 587.
**Hart, W. Malloch**, p. 984.
**Hartley, J. V. J.**, p. 1147.
**Hartwell, John A.**, p. 1058.
**Hautant, A.**, p. 176.
**Hazlett T. Lyle**, p. 1444.
**Head, Henry**, p. 71.
**Hebert, Pierre**, p. 642.
**Heitz-Boyer**, p. 157.
**Henderson, D. K.**, p. 231.
**Henderson, M. S.**, p. 1565.
**Herrold, Russell D.**, p. 638.
**Hewlett, A. M.**, p. 604.
**Hinton**, p. 587.
**Hoffmann, Frederick L.**, p. 1436.
**Hollande**, p. 1567.
**Holmes, Gordon**, p. 53.
**Horsley, J. Shelton**, p. 218.
**Hulton-Frankel, Florence**, p. 1510.
**Hunter, William**, p. 1107.

**Ibbotson, William**, p. 1553.
**Imbert, Léon**, p. 1562.
**Ingraham, C. B.**, p. 1400.
**Interallied Surgical Conference**, p. 57.
**Ireland**, M. W., pp. 655, 255.
**Irvine, H. G.**, p. 1533.
**Ivens, Frances**, p. 1148.

**Jackson**, p. 587.
**Jambon, A.**, p. 1154.
**James, Percy L.** p. 199.
**Jevons, W.**, p. 1049.
**Jobson T. B.**, p. 101.
**Joly**, p. 605.
**Jones, Daniel-Fiske**, p. 1498.
**Joscelyne, F. P.**, p. 327.
**Joslin, M. C.**, p. 577.

**Kahn, I. S.**, p. 1407.
**Karajonopolos**, p. 1145.
**Karsner, H. T.**, p. 1435.
**Kazarjam**, p. 1370.
**Kean, J. R.**, p. 556.
**Kennedy, Foster**, p. 26.
**Kenneway, E. L.**, p. 1523.
**Knowles, Frank Crozer**, pp. 396, 237, 1540.
**Kolmer, John A.**, pp. 614, 634.
**Kouindjy, P.**, p. 1489.
**Krumbhaar, E. B.**, p. 1383.
**Laborde, Simon**, p. 838.
**Lacroix, M.**, p. 1548.
**Laignel-Lavastine**, pp. 44, 751.
**Lambert, Alexander**, pp. 108-116.
**Lambert, A. C.**, p. 1111.
**Lane, Arbuthnot**, p. 1060.
**Lane, F. F.**, p. 1416.
**Lapeyre**, p. 841.
**Laroche, Guy**, pp. 642, 1503.
**Lawson, G. B.**, p. 1427.
**Leconte**, p. 585.
**Lee, Harry**, p. 1577.
**Lee, W. E.**, p. 1150.
**Le Fort, René**, pp. 217, 852.
**Legendre**, p. 874.
**Legrain, P.**, pp. 209, 846.
**Lemaitre, René**, pp. 206, 790.
**Lemere, H. B.**, p. 1159.
**Lemière**, p. 1569.
**LePrince, J. A.**, p. 1112.
**Leriche**, pp. 153, 868, 1063.
**Lessig, John O.**, p. 1576.
**Letarget, M.**, p. 865.
**Levy, Robert L.**, M. C., p. 560.
**Lewis, E. R.**, p. 1163.
**Lhermitte**, p. 1452.
**Lian, Camille**, p. 1439.
**Lilienthal, Howard**, pp. 1480, 1485.
**Little, E. Muirhead**, p. 87.
**Livermore, A. R.**, p. 1575.
**Lochon**, p. 608.
**Lockwood, A. L.**, pp. 6, 293.
**Lollini, A.**, p. 1090.
**Longcope, Warfeld**, p. 566.
**Lucke, Baldwin**, p. 632.
**Lusk, F. B.**, p. 646.
**Lynch, Charles**, p. 619.
**Lyon, G.**, p. 1113.

**McCaw, W. D.**, p. 656.
**McClelland, J. E.**, p. 1397.

McCollum, Neil H., p. 1576.
McCord, C. P., p. 1390.
McDonald, W. W., p. 234.
McFarlane, Andrew, p. 1440.
McGrigor, D. B., p. 1542.
McGuire, L. W., p. 1395.
McIntosh, James, p. 1519.
McKee, S. Hanford, pp. 1158, 1548.
McKenna, Hugh, p. 647.
McNee, J. W., pp. 171, 1519.
MacAdam, W. M., p. 1511.
MacCallum, W. C., p. 623.
MacDonald, R. C., p. 657.
Macdonald, W. M., p. 1413.
MacIlwaine, J. B., p. 224.
MacKenzie, Kenneth, A. J., p. 1138.
MacKinstry, W. H., p. 1408.
Macklin, C. C., p. 381.
Mac-Lean, H., p. 1115.
Magoun, J. A. H., p. 79.
Maingot, G., p. 1544.
Mairet, A., p. 1454.
Mantoux, Ch., p. 1544.
Marie, Pierre, p. 56.
Marion, G., p. 372.
Marlow, S. B., p. 1399.
Martin, p. 90.
Martin, C. J., p. 600.
Martin, M. A., p. 1064.
Martín, Walton, p. 1463.
Masson, J. C., p. 67.
Mathers, George, p. 638.
Mauclaire, p. 1133.
Maude, A., p. 600.
Maxwell, E. M., p. 1549.
Mayers, Lawrence H., p. 626.
Mayet, H., p. 847.
Meader, p. 587.
Meek, p. 587.
Melis, p. 1091.
Mercadé, p. 1136.
Merle, P., p. 1136.
Merrill, A. B., p. 1505.
Metcalf, Carleton R., p. 1496.
Meuriot, pp. 850, 1452.
Meyer, Willie, p. 1065.
Miller, Julius A., p. 1494.
Miller, J. L., p. 648.
Miller, S. R., p. 1093.
Millier, Hugh., p. 1425.
Moncany, p. 874.
Monod, Octave, p. 324.
Moreau, p. 1066.
Moss, p. 347.
Mouchet, p. 871.
Moulonguet, A., pp. 209, 846.
Murlin, J. R., p. 1165.
Musser, J. H., p. 980.
Nageotte, M. J., p. 848.
Neff, p. 757.
Neill, M. H., pp. 135, 1528.
Netter, p. 1098.
Neuhorf, Harold, p. 1458.
Neve, Arthur, p. 870.
Nevin, Mary, p. 1514.
New, G. B., p. 1565.
Nichols, H. J., p. 1555.
Nicoll, James H., p. 1495.
Nifong, F. G., p. 1067.
Nodine, Alonzo Milton, pp. 1370, 1578.
Norris, p. 722.
Novitsky, J., p. 1572.

O'Malley, John F., p. 1556.
Ombredanne, L., p. 361.
Orcutt, Marion L., p. 1506.
Osler, William, p. 1116.

Pacchioni, Dante, p. 639.
Page, G. B., p. 238.
Pamart, p. 871.
Parrott, Arthur H., p. 1559.
Peakock, A. H., p. 1537.
Penfield, W. B,, p. 1521.
Pépin, G., p. 1117.
Perkins, Joseph A., p. 1520.
Peters, John T., 1528.
Philipps, E. V., p. 1049.
Phocas, M., p. 875.
Pickerill, H. P., p. 857.
Pieron, H., p. 1454.
Platon, p. 850.
Plotz, H., p. 1419.
Poe, G., p. 191.
Policard, A., p. 1063.
Pont, 1572.
Potherat, E., p. 866.
Pratt, Joseph H., p. 1096.
Primerose, Alexander, p. 1069.
Proust, p. 196.
Pusey, W. A., p. 1535.
Putte, p. 90.
Putti, V., p. 376.

Qualls, L. p. 1155.
Qualn, E. P., p. 1487.
Quigley, William J., p. 630.

Radin, M. J., p. 1118.
Real, P., p. 1562.
Redden, W. R., p. 1395.
Reilly, J., p. 1432.
Reimann, S. P., p. 79.
Rhein, John H. W., p. 47.
Richards, Owen, p. 78.
Richards, p. 293.

**Richardson**, p. 587.
**Richet, C.**, p. 1071.
**Riddoch, George**, p. 71.
**Rigaux, M. F.**, p. 631.
**Rimmer, R.**, p. 1437.
**Ripert**, pp. 90, 1091.
**Rist**, pp. 348, 972, 998.
**Ritchie, T. R.**, p. 1413.
**Roberts, E. D.**, p. 1451.
**Roberts, John B.**, pp. 1473, 1560.
**Robertson, C. M.**, p. 1120.
**Robertson, H. E.**, pp. 228, 994.
**Robertson, Oswald H.**, p. 68.
**Roddy, J. A.**, p. 1400.
**Rogers**, p. 587.
**Rosanoff, A. J.**, p. 1406.
**Rousseau**, p. 834.
**Roussy, G.**, p. 37.
**Rouvillois**, p. 859.
**Rudolph, R.** p. 1418.
**Russell, F. F.**, p. 1198.

**Saint-Girons, Fr.**, p. 1071.
**St. John, Fordyce Barker**, p. 1458.
**Salmon**, p. 34.
**Sanborn, J. W.**, p. 1575.
**Sard, J. de**, p. 1530.
**Sargent, Percy**, p. 1490.
**Savariaud**, p. 843.
**Sencert, L.** pp. 840, 1133.
**Senn, Nicholas**, p. 106.
**Sergent**, p. 972.
**Shattuck, G. C.**, p. 1050.
**Shaw, H. A.**, p. 656.
**Shaw-Mackenzie, J. A.**, p. 645.
**Sherry**, p. 587.
**Short, J. T.**, p. 1403.
**Siler J.-F.**, p. 348.
**Simonin**, p. 1156.
**Sinclair**, p. 145.
**Sladen, F. G.**, p. 1390.
**Small, A. A.**, p. 624.
**Smith, Clinton K.**, p. 1532.
**Smith, F. M.**, p. 1438.
**Sneyd, George C.**, p. 86.
**Soltau, P. B.**, pp. 293, 320, 1.
**Somerville, W. A.**, pp. 980, 985.
**Speed, Kellogg**, p. 83.
**Spruitt, Charles**, p. 619.
**Squires, W. A.**, p. 1570.
**Steinfield, Edward**, pp. 614, 934.
**Stevens, A. Raymond**, p. 1528.
**Stewart, R. N.**, p. 388.
**Stoddard, J. L.**, p. 1517.
**Stodel, G.**, p. 835.
**Stookey, Byron**, p. 1140.
**Stopford, John S. B.**, p. 232.
**Strathy, G. S.**, p. 1420.
**Sweet, J. E.**, pp. 358, 1121.
**Symmers, D.**, p. 1398.
**Symns, J. L. M.**, p. 1446.
**Symonds, C. P.**, p. 1392.

**Tanton, M. J.**, pp. 80, 235, 868.
**Tarnowsky, G. de**, p. 1500.
**Taylor, F. E.**, p. 1408.
**Taylor, Gordon**, p. 215.
**Taylor, Herbert D.**, pp. 830, 832, 833.
**Taylor, Kenneth**, p. 109.
**Taylor, P. D.**, p. 1151.
**Terhune, W. B.**, p. 99.
**Terrien, F.**, p. 1159.
**Thayer**, pp. 322, 580, 657, 974.
**Thévenot**, p. 839.
**Thibierge, George**, p. 1538.
**Thomas, André**, p. 1351.
**Thompson, James E.**, p. 1492.
**Thompson, Loyd**, p. 1157.
**Thompson, R. S. C.**, pp. 194, 587.
**Thurlow, Madge DeG.**, p. 381.
**Tibbits, E. S.**, p. 1057.
**Tinel, J.**, p. 209.
**Tribondeau, L.**, pp. 1502, 1509.
**Tridon**, p. 871.
**Trotter, R. B.**, p. 1445.
**Trudeau, F. B.**, p. 650.
**Tuffier**, pp. 16, 293, 839, 1076.
**Turner, Philip**, p. 1072.
**Turner, W. H.**, p. 1150.
**Tzanck, A.**, p. 1154.

**Underhill, F. P.**, p. 1525.
**Upcott, C. H.**, p. 1073.

**Vaidya, S. K.**, p. 1142.
**Valentine, Eugenia**, p. 613.
**Vaughan, V. C.**, p. 978.
**Venable, C. S.**, p. 1478.
**Vernet, M. Maurice**, p. 629.
**Vigot, M.**, pp. 374, 1084.
**Villard, E.**, p. 1134.
**Villéon, E. Petit de la**, pp. 24, 851, 372.
**Vincent**, pp. 608, 835, 1098.
**Violle, H.**, pp. 598, 636.
**Virmeaux**, p. 1503.
**Vitrac, J.**, pp. 845, 1135.

**Walther**, p. 1132.
**Warfield, L. M.**, p. 1123, 1438.
**Warrack, J. S.**, p. 1411.
**Warthin, Alfred S.**, p. 1441.
**Wass, Kilton, J.**, p. 1576.
**Watson, E. M.**, p. 1504.
**Weaver, George H.**, p. 1507.
**Webb, Gerald B.**, pp. 581, 973, 979, 989, 996.
**Weiss, Charles**, p. 634.

**Wheaton, C. L.,** p. 650.
**Wheeler, G. W.,** p. 1390.
**White, William Charles,** p. 1124.
**Wiart,** p. 1135.
**Willems, Ch.,** p. 860.
**Williams, Tom A.,** p. 51.
**Wilmer, H. B.,** p. 1121.
**Wilmer, W. H.,** p. 1160.
**Wilson, A.,** p. 556.
**Wilson, D. W.,** p. 1525.
**Wilson, Philip D.,** p. 218.
**Wilson, S. A. Kinnier,** p. 102.
**Winter, F. A.,** p. 656.
**Wolfsohn, Julian M.,** p. 1449.
**Woodyatt, Rollin T.,** p. 1092.
**Wooler, E. W. N.,** p. 1075.
**Wright, A. M.,** pp. 203, 995, 1513.

**Young, G.,** p. 603.

**Zimmerman, B. F.,** p. 1399.
**Zinsser, H.,** pp. 316, 346.

